# *IL6* gene polymorphism association with calcific aortic valve stenosis and influence on serum levels of interleukin-6

**DOI:** 10.3389/fcvm.2022.989539

**Published:** 2022-10-20

**Authors:** Alejandro Junco-Vicente, Guillermo Solache-Berrocal, Álvaro del Río-García, Valeria Rolle-Sóñora, Sheila Areces, César Morís, María Martín, Isabel Rodríguez

**Affiliations:** ^1^Department of Cardiology, Área del Corazón, Hospital Universitario Central de Asturias (HUCA), Oviedo, Spain; ^2^Cardiac Pathology Research Group, Instituto de Investigación Sanitaria del Principado de Asturias (ISPA), Oviedo, Spain; ^3^Biostatistics and Epidemiology Platform, Instituto de Investigación Sanitaria del Principado de Asturias (ISPA), Oviedo, Spain; ^4^Department of Medicine, Faculty of Medicine, University of Oviedo, Oviedo, Spain

**Keywords:** aortic valve stenosis, calcific aortic valve disease, *IL6*, interleukin-6, *PALMD*, polymorphism

## Abstract

Aortic valve stenosis is the most frequent valve disease in developed countries and its prevalence will increase with population aging. There is still no pharmaceutical treatment nor biomarker to determine the susceptibility to develop aortic stenosis. Therefore, we analyzed the association of polymorphisms in risk loci with calcific aortic stenosis. Patients with aortic valve disease were genotyped for *PALMD* rs6702619, *LPA* rs10455872, and *IL6* rs1800795 polymorphisms and circulating levels of interleukin-6 (IL-6) were measured. Calcium content of leaflets obtained in valve replacement surgeries was determined by micro-computed tomography. In the genotyping of 578 individuals, we found significant association between *PALMD* and *IL6* polymorphisms and aortic stenosis in patients with tricuspid aortic valve, independently of other potentially confounding variables such as age and dyslipidemia. There was no association of these polymorphisms with valve calcium content, but this value correlated with the mean aortic pressure gradient (*r* = 0.44; *P* < 0.001). The CC genotype of *IL6* polymorphism was associated with higher levels of serum IL-6 compared to other genotypes (23.5 vs. 10.5 pg/ml, respectively; *P* = 0.029). Therefore, patients carrying the CC genotype of *IL6* rs1800795 polymorphism present higher levels of circulating IL-6 and this could contribute to the severity of the aortic valve stenosis. Our results agree with the identification of *IL6* as a locus risk for stenosis and also with the intervention of this cytokine in aortic valve calcification. A more exhaustive follow-up of those patients carrying risk genotypes is therefore recommended.

## Introduction

Aortic stenosis is the most frequent valvular disease in developed countries. Among its possible etiologies, the one produced by valve calcification is the most common, behaving as a chronic and progressive entity associated with aging. Given the longer life expectancy in developed countries, its prevalence is increasing and becoming a significant public health problem. There are studies that estimate its incidence will double in 2040 and triple by 2060 (1).

Severe calcific aortic stenosis represents the most advanced stage of calcific aortic valve disease (CAVD) (2). It is a complex pathological process that progresses asymptomatically to advanced stages. When it reaches severity and produces symptoms, the patient’s prognosis is unfavorable and, according to the clinical practice guidelines, valve replacement becomes necessary (3). Actually, replacement should be also considered for patients with moderate aortic stenosis undergoing cardiac surgery for other reasons (3).

Despite its prevalence, morbidity and mortality, to date, no medical treatment for aortic stenosis can ameliorate its natural history and intensive research has not yet been able to discover a therapy capable of stopping or reversing its progression (4). Although many drugs have been proposed as potential treatments against the disease, acting on molecular or cellular processes characteristic of the different stages of progression such as lipid lowering or bone metabolism pathways, none have shown beneficial effects (5–9). At present, there are no effective prevention strategies either. Several studies in the last decade have discovered susceptibility genes for aortic stenosis, albeit in a limited number, as well as some associated polymorphisms. Remarkably, genome-wide association studies (GWAS) involving persons with either computed tomography (CT)-detected valvular calcification or clinical aortic stenosis have enabled the discovery of unexpectedly associated genes like *PALMD*, *NAV1*, *TEX41*, *CACN1C*, and other important genes implicated in its pathophysiology, like *LPA*, *ALPL*, *RUNX2*, and *IL6* (10–13). These genetic polymorphisms could be used to identify individuals at risk, thus enabling preventive strategies to be implemented, while they also contribute to the discovery of new potential therapeutic targets.

The most consistently replicated polymorphisms in previous studies have been those of the *PALMD*, a gene coding for palmdelphin, and *LPA*, coding for apolipoprotein(a). Polymorphisms located in these genes and an additional one in *IL6*, the gene coding for interleukin-6, a cytokine involved in inflammation, were therefore analyzed in our population to test for their association with aortic stenosis and their potential utility in the search for biomarkers and therapeutic targets.

## Materials and methods

### Patients

We analyzed a total population of 734 individuals prospectively recruited in three cohorts, all from the same region in Spain, and through the Cardiology Department of our hospital. Established guidelines and criteria for the diagnosis of aortic stenosis and regurgitation by echocardiographic analysis were used (14). The main diagnosis was based on aortic valve while patients with severe pathology in other heart valves were excluded. Rheumatic valve disease and endocarditis were exclusion criteria for the collection. Valve morphology other than unambiguous tricuspid or bicuspid, and the simultaneous presence of both stenosis and regurgitation (mixed aortic valve disease) (15) were omitted from the analysis. Individuals undergoing an ultrasound procedure showing a normally functioning aortic valve were used as controls for aortic stenosis, together with patients with aortic regurgitation. Anthropometric, demographic, and biochemical data were obtained from medical records, at medical consultation for outpatients, or during pre-surgery visit, according to routine medical practice. Two cohorts were previously described (16, 17). The third one came from a project to prospectively collect all the aortic valve samples obtained in replacement surgeries performed in our hospital between November 2016 and November 2017 (*n* = 336). The study was conducted in accordance with the Declaration of Helsinki and the human sample collection protocols were approved by the Ethics Committee for Investigation of the Principality of Asturias (84/13 and 90/17). All patients signed an informed consent form before enrolment.

### Genotyping

Peripheral blood samples obtained in EDTA tubes were processed to obtain genomic DNA, following standard procedures, and this was stored at −20^°^C. Samples were genotyped by quantitative PCR using Taqman probes (Thermo Fisher Scientific) for the following polymorphisms: rs6702619 of *PALMD* (C_26334289_10), rs10455872 of *LPA* (C_30016089_10), and rs1800795 of *IL6* (-174G > C; C_1839697_20). Genotypes were analyzed with StepOne Software v2.3. Around 5% of the samples were randomly selected and re-genotyped in order to confirm the accuracy of the genotyping procedure. No discrepancies were found.

### Measurement of calcium content in valve leaflets by micro-computed tomography

Tissue samples were preserved in RNAlater (Ambion) in the operating room at the time of surgical valve replacement and were sent to the Biobank for subsequent manipulation. A leaflet from each valve, or half a leaflet from bicuspid aortic valves (BAV), was preserved in 70% ethanol at 4^°^C until use. Calcium content on valve leaflets was measured by micro-computed tomography (microCT) as previously described (18). In brief, ethanol-preserved samples were analyzed in a SkyScan 1174 high-resolution tomograph (Bruker, Billerica, MA, USA). Specimens were scanned at 50 kV of source voltage and 800 μA X-ray tube current. An exposure time of 6,200 ms was used and each scan was taken between 10 and 20 min depending on the size of the leaflet. The amount of calcium deposited was expressed as the ratio between bone volume and total volume of the tissue sample (BV/TV), a parameter commonly used in bone histomorphometry (19).

### Measurement of circulating levels of IL-6

Blood samples to isolate serum were collected at medical consultation in outpatients or pre-surgery in the case of aortic valve replacement. Serum was stored at −80^°^C until use. Circulating levels of interleukin-6 (IL-6) were measured as part of a Human Luminex Discovery Multiplex Assay (R&D Systems) following the manufacturer’s protocol.

### Statistical analysis

Quantitative variables were compared between groups using Mann Whitney’s U and qualitative variables using Fisher test. Hardy-Weinberg equilibrium for the polymorphisms was tested by means a Chi-squared test. Multivariable logistic regression models were used to detect association between polymorphisms and stenosis. The complete model was adjusted with classical cardiovascular risk factors (age, sex, smoking status, hypertension, diabetes mellitus, dyslipidemia), using as the dependent variable diagnosis grouped into two categories: normally functioning controls plus regurgitation vs. stenosis. Odds ratio (OR), 95% confidence interval (CI) and *P*-values of the model are reported. For the analysis involving the BV/TV variable, Mann-Whitney U or Kruskal-Wallis test were used. Correlations between variables were determined using Pearson’s coefficients. A *P*-value < 0.05 was considered statistically significant. Statistical analyses were performed using R software version 4.1.3 (20).

## Results

### *PALMD* rs6702619 and *IL6* rs1800795 polymorphisms are associated with aortic stenosis in tricuspid aortic valve patients

**FIGURE 1:**
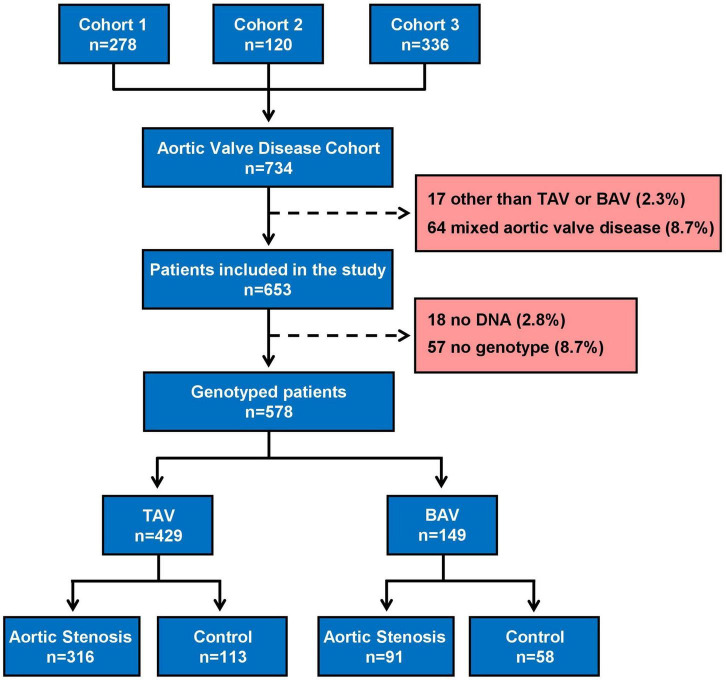
Flowchart of patient selection. BAV, Bicuspid aortic valve; TAV, Tricuspid aortic valve; Control, normally functioning aortic valve plus aortic regurgitation.

The starting population consisted of 734 individuals of European ancestry. Patient selection allowed us to include 653 patients in the study (see flow chart in Figure 1) whose characteristics, grouped them according to the presence or absence of aortic stenosis, are summarized in Table 1. The group of patients with aortic stenosis had mainly severe aortic stenosis. There were no statistical differences in sex or estimated glomerular filtration rate between groups, but the control group was younger and BAV and regurgitation were more frequent. The aortic stenosis group had more patients with hypertension, dyslipidemia, diabetes and coronary artery disease than the control group.

**TABLE 1:** Characteristics of patients included in the study, according to medical diagnosis.

Characteristic	Control (*n* = 175)	Aortic stenosis (*n* = 478)	*P*-value
Age, years (Median [Q1, Q3])	62.0 [52.0, 72.0]	75.0 [66.0, 79.0]	< 0.001
Sex, *n* (%)			0.367
Male	111 (63.4)	283 (59.2)	
Female	64 (36.6)	195 (40.8)	
Aortic valve morphology, *n* (%)			0.002
Tricuspid aortic valve	115 (65.7)	372 (77.8)	
Bicuspid aortic valve	60 (34.3)	106 (22.2)	
Smoking status, *n* (%)			0.002
Current smoker	45 (25.7)	70 (14.6)	
Former smoker	23 (13.1)	95 (19.9)	
Non-smoker	107 (61.1)	313 (65.5)	
Hypertension, *n* (%)			< 0.001
Yes	94 (53.7)	333 (69.7)	
No	81 (46.3)	145 (30.3)	
Dyslipidemia, *n* (%)			< 0.001
Yes	64 (36.6)	267 (55.9)	
No	111 (63.4)	211 (44.1)	
Diabetes mellitus, *n* (%)			0.005
Yes	32 (18.3)	139 (29.1)	
No	143 (81.7)	339 (70.9)	
Regurgitation, *n* (%)			< 0.001
Yes	100 (57.1)	64 (13.4)	
No	75 (42.9)	414 (86.6)	
eGFR, mL/min/1.73 m^2^ ^a^	73.0 (19.9)	69.2 (20.1)	0.383
Coronary artery disease, *n* (%)			0.015
Yes	6 (12.5)	97 (29.0)	
No	42 (87.5)	237 (71.0)	
Missing	127 (72.6)	144 (30.1)	
**Echocardiography**			
Mean Gradient, mmHg^b^	17.1 (13.7)	48.2 (12.0)	< 0.001
Peak Gradient, mmHg^c^	30.9 (21.7)	79.9 (18.5)	< 0.001

Quantitative variables are presented as mean (standard deviation) unless otherwise specified. Qualitative variables are presented as frequency (percentage). eGFR, Estimated glomerular filtration rate.

^a^Data available for 382 patients.

^b^Data available for 292 patients.

^c^Data available for 272 patients.

The whole population fitted the Hardy-Weinberg equilibrium for the three polymorphisms. The comparison of genotype and allele frequencies, age and sex adjusted, yielded significant differences between case and control groups for *IL6* rs1800795 polymorphism in the whole population (Table 2, OR 2.11, 95% CI 1.07–4.40, *P* = 0.038 for CC genotype and OR 1.37, 95% CI 1.02–1.83, *P* = 0.035 for each C allele). Stratifying by aortic valve morphology, *PALMD* rs6702619 and *IL6* rs1800795 polymorphisms were associated with aortic stenosis in tricuspid aortic valve (TAV) patients only (Table 2). In accordance with the model of inheritance, the *PALMD* polymorphism follows a recessive pattern, since the effect is only statistically significant when the minor allele is in homozygosity (GG genotype, OR 1.99, 95% CI 1.01–3.97, *P* = 0.049). In contrast, an additive model can be applied to *IL6* polymorphism, taking into account the increasing effect (OR) of CC (OR 4.73, 95% CI 1.61–17.90) compared to CG genotype (OR 1.68, 95% CI 1.02–2.78) (Table 2). *LPA* rs10455872 polymorphism was not associated with aortic stenosis, although the statistical power to detect an association was limited by the low frequency of the minor allele in our population (9%).

**TABLE 2:** Association between polymorphisms and aortic stenosis according to aortic valve morphology.

		All (*n* = 578) (70% aortic stenosis)		TAV (*n* = 429) (74% aortic stenosis)		BAV (*n* = 149) (61% aortic stenosis)	
Polymorphism	Genotype/Allele	OR, 95% CI	*P*-value	OR, 95% CI	*P*-value	OR, 95% CI	*P*-value
*PALMD* rs6702619	GT	1.34, 0.82–2.19	0.243	1.37, 0.74–2.49	0.311	1.57, 0.59–4.09	0.359
	GG	1.58, 0.92–2.73	0.097	1.99, 1.01–3.97	0.049	1.05, 0.38–2.86	0.927
	G	1.26, 0.96–1.65	0.100	1.42, 1.01–2.00	0.045	0.95, 0.58–1.56	0.846
*LPA* rs10455872	AG	1.14, 0.67–2.01	0.635	1.26, 0.65–2.53	0.506	0.94, 0.32–3.02	0.919
	GG	4.20, 0.55–88.82	0.226	6.05, 0.23–319.2	0.357	NA, 0.00–NA	0.988
	G	1.31, 0.80–2.22	0.295	1.36, 0.74–2.64	0.343	1.42, 0.54–4.13	0.493
*IL6* rs1800795	CG	1.30, 0.87–1.95	0.206	1.68, 1.02–2.78	0.043	0.73, 0.34–1.55	0.412
	CC	2.11, 1.07–4.40	0.038	4.73, 1.61–17.90	0.010	0.65, 0.24–1.81	0.404
	C	1.37, 1.02–1.83	0.035	1.72, 1.19–2.52	0.005	0.77, 0.46–1.28	0.309

OR, odds ratio; CI, confidence interval; TAV, tricuspid aortic valve; BAV, bicuspid aortic valve; NA, not available because in the Control group there were no GG individuals. Allele means risk allele. Reference genotype for *PALMD*, TT; for *LPA*, AA; for *IL6*, GG. *P*-values after age and sex adjustment.

**FIGURE 2:**
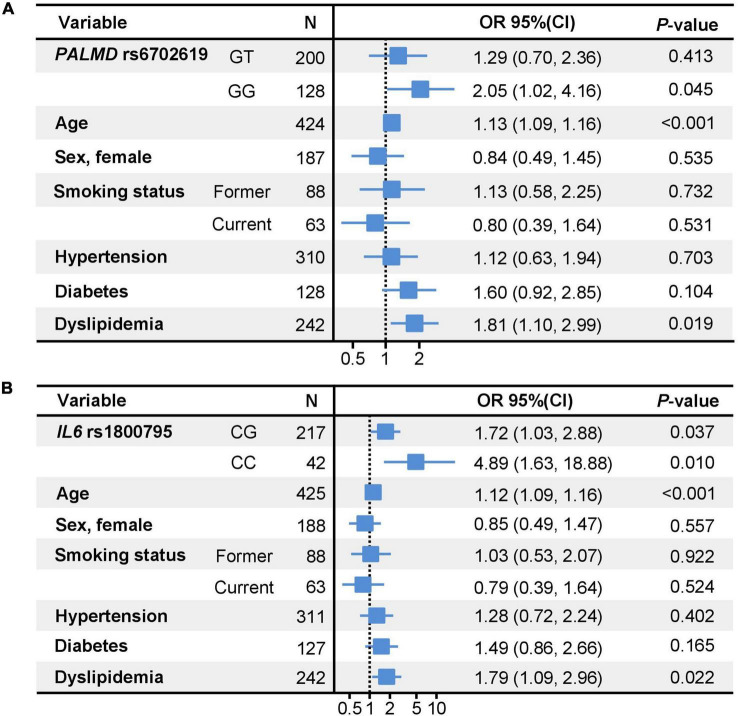
Forest plots showing the multivariable logistic regression models for the association between *PALMD* **(A)** and *IL6* **(B)** genotypes and aortic stenosis. Reference for smoking status: never smoked. Reference for *PALMD* rs6702619 polymorphism **(A)**: TT genotype. Reference for *IL6* rs1800795 polymorphism **(B)**: GG genotype; OR, Odds Ratio; CI, Confidence Interval.

Focusing the analysis on TAV, we confirmed the association of these two polymorphisms in *PALMD* and *IL6* with aortic stenosis, independently of potentially confounding variables such as age and dyslipidemia (Figure 2).

### Association of polymorphisms with other cardiovascular traits

We then analyzed the association of these polymorphisms with several cardiovascular traits, some of them acting as potential risk factors for aortic stenosis. We found a significant association between *LPA* polymorphism and coronary artery disease in the subset of 382 participants (334 cases and 48 controls) for which this information was available, with OR 2.30, 95% CI 1.35–3.94, *P* = 0.003, per risk allele. In addition, a borderline association was also found for *IL6* polymorphism and both coronary artery disease and hypertension (Table 3).

**TABLE 3:** Association analyses between polymorphisms and several cardiovascular traits.

Characteristic (*n* = 578)	*PALMD* rs6702619		*LPA* rs10455872		*IL6* rs1800795	
	OR, 95% CI	*P*-value	OR, 95% CI	*P*-value	OR, 95% CI	*P*-value
Bicuspid aortic valve	0.83, 0.63–1.08	0.176	0.72, 0.44–1.18	0.237	0.99, 0.75–1.31	1.000
Regurgitation	0.88, 0.67–1.16	0.402	0.92, 0.57–1.49	0.809	1.05, 0.79–1.40	0.772
Hypertension	0.99, 0.77–1.26	0.951	0.68, 0.45–1.03	0.069	1.28, 1.00–1.64	0.055
Dyslipidemia	0.96, 0.76–1.21	0.767	1.40, 0.93–2.11	0.121	0.97, 0.76–1.24	0.853
Diabetes mellitus	0.90, 0.69–1.18	0.497	0.98, 0.61–1.56	1.000	0.88, 0.67–1.16	0.358
Coronary artery disease^a^	1.21, 0.85–1.72	0.320	2.30, 1.35–3.94	0.003	1.46, 1.00–2.13	0.051

OR, odds ratio; CI, confidence interval.

^a^Data available for 382 patients. Risk allele for *PALMD*, G; for *LPA*, G; for *IL6*: C.

### Association of polymorphisms and aortic valve calcium

Since the association of *PALMD* and *IL6* polymorphisms were specific for stenosis in TAV, we next analyzed whether there was an association between the polymorphisms and valve calcium content. Thus, 82 valve leaflets from TAV patients (77 from stenosis patients and 5 from regurgitation patients) were analyzed by microCT. As expected (18), calcium levels measured as BV/TV were significantly higher in stenotic valves [median (range): 3.46% (11.89) for stenotic vs. 0.10% (1.26) for regurgitant valve, *P* < 0.001]; however, regarding the association of the polymorphisms with the extent of calcification, no statistically significant differences were found for BV/TV values between the different genotypes (*P* = 0.476 and *P* = 0.197, for *PALMD* and *IL6*, respectively). Even comparing BV/TV levels for homozygotes of the risk allele (GG genotype for *PALMD* and CC genotype for *IL6*) with the levels of other genotypes, after adjustment by diagnosis of aortic stenosis, the differences were not statistically significant (Supplementary Table 1). There was also no association between the calcium content of the valve and the *LPA* polymorphism, in this case grouping the homozygotes of the risk allele with the heterozygotes due to the low number of the former (Supplementary Table 1). There was, instead, a positive correlation between BV/TV values and mean aortic pressure gradient, which is one of the primary hemodynamic parameters recommended for clinical evaluation of aortic stenosis severity (Pearson’s coefficient *r* = 0.44, *P* < 0.001).

### *IL6* genotype is associated with circulating levels of IL-6

**FIGURE 3:**
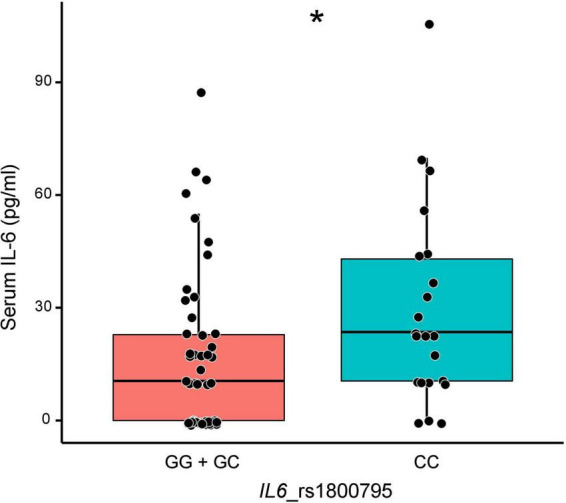
Levels of circulating interleukin-6 (IL-6) according to *IL6* rs1800795 genotypes. *P = 0.006.

We next investigated whether the different genotypes could have a reflection on the levels of the respective proteins in order to use them as biomarkers. To our knowledge, PALMD is not released to the circulation.^1^ By contrast, we investigated whether the *IL6* polymorphism could have an effect on the circulating levels of the cytokine in our patients, as it is described for coronary artery disease (21), by measuring IL-6 levels in 76 serum samples selected according to rs1800795 genotype. The CC genotype was associated with higher levels of serum IL-6 (23.5 pg/ml vs. 10.5 pg/ml for the other genotypes; *P* = 0.006) (Figure 3), even after adjustment for age, hypertension and coronary artery disease (estimate: 13.92, 95% CI 2.16–25.68, *P* = 0.021). IL-6 levels were not different between stenosis and regurgitation patients (10.5 vs. 14.4 pg/ml, respectively; *P* = 0.864). In addition, IL-6 levels positively correlated with levels of the inflammatory marker C-reactive protein (CRP; Pearson’s coefficient *r* = 0.83, *P* < 0.001).

## Discussion

Pharmacological therapies for aortic stenosis continue to be an important target of biomedical research, as they could benefit a very large group of people. The reasons why some individuals develop aortic stenosis over time while others do not are also unknown due to the complex genetic and molecular processes that produce this disease. The identification of individuals with a genetic predisposition for the development of the disease is essential for the benefit of closer medical follow-up. In our study, we have confirmed that polymorphisms in *PALMD* and *IL6* genes are associated with aortic valve stenosis and the latter one, in addition, with higher circulating levels of IL-6.

The rs1800795 polymorphism in the promoter region of *IL6* gene is one of the most widely analyzed in cardiovascular diseases. A recent meta-analysis examined more than 40 studies related to rs1800795, with the firm conclusion that there is a clear association with coronary artery disease driven, at least in part, by upregulating plasma IL-6 levels (21). The hypothesis in that study is that carriers of the C allele, through various mechanisms not yet fully clarified, have upregulated transcription and translation of the gene. Therefore, individuals with both C alleles present higher concentrations of circulating IL-6, as we have demonstrated in our population, putting them at higher risk of developing atherosclerosis-like diseases. However, in our population, we did not find higher levels of circulating IL-6 in aortic stenosis patients, despite the higher frequency of this allele in these patients. Investigating and determining the effect of a single polymorphism is not straightforward, due to the multiple interactions with other polymorphisms and molecular synergisms that may underlie phenotypic changes (22). Perhaps other *IL6* polymorphisms and epigenetic modulation should be taken into account to better explain the genetic regulation of IL-6 levels which has recently been proven to be very complex (23). More information is needed in the literature about haplotypic data of *IL6* and its relationship with cardiovascular disease.

The cytokine IL-6 is a circulating pro-inflammatory peptide produced by many different cell types, including lymphocytes, monocytes, fibroblasts, and endothelial cells (24). It has been shown that a high expression of IL-6 in the aortic valve promotes mineralization (25), however, we have not found a correlation between levels of circulating IL-6 and calcium content in the valve, measured by microCT as BV/TV. *In vitro* studies have instead demonstrated the relationship of IL-6 with bone morphogenetic protein 2 (BMP-2) and RUNX2, important regulators and promoters of osteogenesis and key elements in the calcification process by controlling the osteogenic transition of valvular interstitial cells during CAVD (25). IL-6 can promote as well the endothelial to mesenchymal transition of valvular endothelial cells, suspected to be one of the first stages of the calcification process of the valve leaflets (26). These findings led to the attempt to treat aortic stenosis with drugs designed to control calcification, although some of them, such as denosumab or alendronic acid, did not affect progression of aortic valve calcification in patients with calcific aortic stenosis (9). There are still some ongoing clinical trials of drugs that attempt to control this calcification pathway and others that have been implicated in CAVD (NCT03305536, NCT04429035, NCT04055883, NCT03051360). However, the repeated failures in treatments highlight the need to continue investigating the molecular pathways that lead to valve calcification in order to find new therapeutic targets (4, 27).

Calcium deposition generates valve stiffness that reduces the valve opening, thus obstructing the outflow of the left ventricle, and therefore, increasing the speed of blood flow through it. Thus, when velocity is measured by echocardiography, the transvalvular gradient and the area serve to grade the stenosis. When these parameters raise doubts about the severity, the use of a calcium-score (CT quantification of valvular calcium levels) has become widespread in clinical practice in recent years to help in classifying the degree of severity of aortic stenosis (28, 29). In our study, the calcium levels measured in the explanted tissue (valve leaflets) showed a congruent and positive correlation with the valve gradient measured by ultrasound. However, there was no significant association of the extent of calcification with the *IL6* polymorphism, suggesting that the influence of the polymorphism in the disease is related to another feature or is limited to initial stages of the disease, considering that most of our patients had advanced severe aortic stenosis.

The principal action of IL-6 is to stimulate the synthesis of all the acute phase proteins involved in the inflammatory response. This role has been extensively studied in coronary artery disease and cardiac fibrosis (30, 31). The onset of CAVD occurs many years before reaching the state of severity and it is believed that the progressive fibro-calcification presents an initial stage related to disrupted endothelium, lipid deposits and inflammation in the leaflets analogous to the process of atherosclerosis (32). In fact, up to 15% of the more than 700 differentially regulated genes in CAVD compared to a healthy valve are related to inflammation (33). Histological studies revealed as well that inflammation is a mechanism linked to the calcification of aortic valves (34) and a higher expression of IL-6 has already been described in that tissue (25). We have demonstrated, in a subgroup of patients, elevated CRP serum levels correlating with IL-6 serum levels, in agreement with results in other pathologies (35). We cannot state whether this correlation is cause or consequence of the valve calcification since our measurements were made with aortic stenosis already established but still, the result supports the importance of considering molecular inflammatory targets for CAVD treatment. The quantification of additional circulating proteins will contribute to complete the inflammatory profile of these patients that could lead to the identification of biomarkers, single or multi-marker, in order to complement the clinical evaluation (36).

The genetic part of our study also included the analysis of the association of two other polymorphisms that had already been associated with aortic stenosis. The rs6702619 polymorphism related to the *PALMD* gene has been associated with CAVD in two GWAS carried out in recent years (11, 37) and it was also associated in our study. The genetic association was demonstrated first and only recently the protein was implicated in the pathological process in relation to the protection of valvular endothelial cells against mechanical stress but, also, through its involvement in an inflammatory mechanism (38, 39). In addition, the rs6702619 polymorphism was demonstrated to be located on a distal-acting enhancer of *PALMD* gene, affecting the regulation of the gene and promoting fibrosis, which is another pathological process involved in CAVD (40). Therefore, PALMD could be a suitable candidate to be considered as part of a risk prediction model for CAVD and as a potential therapeutic target.

There was a lack of association of CAVD with the rs10455872 polymorphism on *LPA* gene in our population but we replicated the association of this polymorphism with coronary artery disease. This gene encodes apolipoprotein(a), which is a component of the lipoprotein(a) (41). Elevated plasma levels of lipoprotein(a) are a well-known risk factor for cardiovascular disease and are also genetically determined. In fact, a causal association between elevated levels of lipoprotein(a) and increased risk of myocardial infarction or coronary artery disease has been demonstrated (42, 43). Several GWAS have replicated an association between this *LPA* polymorphism and aortic stenosis (10, 12, 37). Nevertheless, in some of the subsets included in the meta-analysis by Helgadottir et al. there was no significant association, specifically in the population of Sweden (Stockholm) and that of the USA (Michigan) (37). Notably, the two populations are the ones with much fewer participants, highlighting the importance of sample size in obtaining statistically significant results, especially for small effects. This can be the case in our population, although we cannot rule out that these different results may be due to different criteria when selecting the populations, such as inclusion or exclusion of individuals with BAV, with regurgitation or with coronary artery disease. In addition, the criteria for selection of the group with aortic stenosis may be based on the quantification of valvular calcium by CT, on the clinical diagnosis based on echocardiography, or on having undergone aortic valve replacement, leading to different findings. Interestingly, elevated lipoprotein(a) levels and corresponding *LPA* risk genotypes have also been associated with increased risk of aortic valve stenosis in two large studies with general population (44, 45). One noteworthy difference with our study, apart from the overall population size, is the higher mean age of our stenosis group because in these studies the cases were incident stenosis and, therefore, relatively younger than our patients, who mostly had advanced severe stenosis. This might detract from the primary role of genetics on the development of stenosis in our older age group.

### Study limitations

There are some limitations to the study and the interpretation of our data. First, the number of participants with normally functioning aortic valve was low and, therefore, the control group included mainly patients with aortic valve regurgitation. A proportion of patients in the case group also suffered from regurgitation but only to a mild degree, whereas regurgitation in the control group was moderate or severe. However, to the best of our knowledge, no association has been found between any of these polymorphisms and aortic regurgitation and, therefore, this characteristic probably does not interfere in the association with aortic stenosis. Second, the number of tissue samples from patients with regurgitation was low, although calcium levels were very consistent and toward a null value, since all of them are expected to be free of calcium. Third, we do not have all the echocardiographic details in the quantification of the severity of the stenosis, nor was CT calcium-score performed in the patients. Finally, potential unmeasured confounders might affect the association between the polymorphism and the presence of aortic stenosis.

## Conclusion

Since we are dealing with a polymorphism that influences the expression of IL-6, our results not only agree with the identification of *IL6* as a risk locus for stenosis, but also with the involvement of this cytokine in valvular calcification. The CC genotype of the rs1800795 polymorphism of the *IL6* gene is associated with calcific aortic stenosis and, in addition, patients with this genotype have higher levels of circulating IL-6. The latter characteristic is a risk factor that may dispose to greater susceptibility to progressive inflammation and calcification processes, worsening their prognosis. Our results serve as a basis for recommending a more exhaustive follow-up of those patients. The fundamental determinants of the disease could be several common genetic variants, each of them leading to a slight increase in the risk. Further studies replicating associations of additional discovered associated variants are required to complete the genetic landscape of this disease.

## Data availability statement

The original contributions presented in the study are included in the article/Supplementary material, further inquiries can be directed to the corresponding author/s.

## Ethics statement

The studies involving human participants were reviewed and approved by Ethics Committee for Investigation of the Principality of Asturias. The patients/participants provided their written informed consent to participate in this study.

## Author contributions

MM, IR, and CM conceived and designed the study. AJ-V and SA contributed to acquisition of clinical data. GS-B and ÁR-G performed the experiments. AJ-V, GS-B, ÁR-G, VR-S, IR, and MM contributed to data analysis and interpretation. All authors contributed to writing and reviewing the manuscript and provided approval for publication of the content.

## Footnotes

1
https://www.proteinatlas.org/ENSG00000099260-PALMD/tissue
