# Supplementary material for: IL6 gene polymorphism association with calcific aortic valve stenosis and influence on serum levels of interleukin-6
Source: Front Cardiovasc Med. 2022 Oct 20;9:989539. doi: 10.3389/fcvm.2022.989539 (PMC9630837; doi:10.3389/fcvm.2022.989539)
Supplement: Supplementary file 1 [file Table_1.DOCX]

Supplementary Material

# Supplementary Table

Association between *PALMD*, *LPA* and *IL6* polymorphisms and aortic valve calcium content measured by microcomputed tomography.

| **Polymorphism** | **Genotype** | **BV/TV** | ***P*-value** |
| --- | --- | --- | --- |
| ***PALMD* rs6702619** | GG (n=28) | 3.29% [4.56] | 0.660 |
|  | GT+TT (n=36+18) | 3.38% [5.05] |  |
| ***LPA* rs10455872** | GG+GA (n=14+1) | 2.74 [3.99] | 0.608 |
|  | AA (n=67) | 3.51 [4.53] |  |
| ***IL6* rs1800795** | CC (n=20) | 4.59% [5.25] | 0.161 |
|  | CG+GG (n=35+27) | 3.28% [4.13] |  |

BV/TV: bone volume/tissue volume, median [interquartile range]. *P*-value after adjustment by diagnosis of aortic stenosis.
